# Impact of longer working hours on fathers’ parenting behavior when their infants are 6  months old: The Japan Environment and Children’s Study

**DOI:** 10.3389/fpubh.2023.1100923

**Published:** 2023-06-27

**Authors:** Haruka Kasamatsu, Akiko Tsuchida, Kenta Matsumura, Kei Hamazaki, Mariko Inoue, Hidekuni Inadera, the Japan Environment and Children’s Study Group

**Affiliations:** ^1^Toyama Regional Center for JECS, University of Toyama, Toyama, Japan; ^2^Department of Public Health, Faculty of Medicine, University of Toyama, Toyama, Japan; ^3^Department of Public Health, Gunma University Graduate School of Medicine, Maebashi, Japan

**Keywords:** birth cohort, childrearing and child care, long working hours, play, work-style reform

## Abstract

**Objective:**

Long working hours have been suggested to affect fathers’ parenting behavior, but previously reported findings have been inconsistent. This study examined the association between the working hours and parenting behavior of fathers while accounting for other factors related to their parenting behavior, using data from the Japan Environment and Children Study (JECS), a large cohort study in Japan.

**Methods:**

Data from 43,159 father–mother pairs were analyzed. The mother assessed the father’s frequency of seven parenting behaviors at 6 months after delivery. Then, each behavior was classified into a high-engagement group (always and sometimes) or a low-engagement group (rarely and never). The father’s weekly working hours was obtained from his responses and was classified into six levels.

**Results:**

Logistic regression analysis showed that after adjustment for covariates, fathers’ weekly working hours was inversely associated with the frequency of all parenting behaviors examined in this study (*p* for trend <0.0001). Compared with fathers working ≥0 to ≤40 h per week, those working >65 h per week showed the following adjusted odds ratios (95% confidence intervals) for low engagement in parenting behaviors: playing at home, 2.38 (2.08–2.72); changing diapers, 2.04 (1.89–2.20); and bathing the child, 2.01 (1.84–2.18).

**Conclusion:**

This study suggests that the greater time constraints imposed by longer working hours constitute a major factor that discourages fathers from engaging in childrearing behavior. Intervention targeting long working hours could contribute to measures aimed at promoting high-engagement parenting behaviors among fathers.

## 1. Introduction

Previous studies have reported many benefits of fathers’ early involvement with their children in the postpartum period, including children’s accelerated neurodevelopment (1) and positive behavioral outcomes at school age (9 and 11 years) (2), as well as reduced risk of mothers’ psychological distress at 1 year after delivery (3). In recent years in the United States and Europe, the traditional image of fathers as the breadwinner has evolved into that of “involved fathers,” “new fathers,” and “new men” to also include their role in caring for their children and in family life more generally (4–6). Time-use surveys conducted in 20 countries between 1965 and 2003 (7) have shown that men’s unpaid work time, including child care, has increased over time.

In relation to this trend, studies have investigated factors associated with fathers’ childrearing behavior, mostly since the 1990s. From a sociological perspective, Shelton et al. reported five factors determining fathers’ engagement in housework and childrearing: time availability, household demands, relative resources, ideology, and alternative resources (8). Empirical studies have examined the relationship of fathers’ childrearing behavior with various factors, including working hours and employment status (9–11), mothers’ educational background (12), age of the youngest child and number of children (13), mothers’ income as a proportion of total family income (13, 14), essentialist perceptions of men and women as parents (15), and ethnicity and country context (12, 16).

Among these factors, fathers’ working hours has been studied in the United States and Europe, Korea, Singapore, Australia, Israel, and Japan as an indicator that can be objectively measured in any country in order to consider individual fathers’ time availability. Although many studies have found an inverse association between fathers’ work hours and frequency of parenting behaviors (13, 15, 17, 18), others have reported a limited association (12, 14), an association weakened by other factors (9), or no association (5, 19, 20), so the strength of association is inconsistent among previous studies. In addition, research on fathers’ parenting behavior and its determinants does not have as long a history as such research on mothers (4). Most studies to date have involved hundreds to thousands of participants, and only a few studies have involved tens of thousands of participants.

Among the countries where previous studies on fathers’ working hours have been conducted, in Japan, men tend to work longer hours compared with men in other Organisation for Economic Co-operation and Development (OECD) countries, at an average of 451.8 min per day compared with 317.8 min per day. Meanwhile, the average time spent by Japanese men on unpaid work at home, including housework and childcare, is 40.8 min per day, which is less than one-third of the 136.5 min per day for men in other OECD countries. Japanese women, in comparison, spend an average of 224.3 min per day engaged in unpaid work at home, highlighting the still large burden of housework and childcare that falls on mothers (21). One possible explanation for the inconsistency in the association between fathers’ working hours and the frequency of childrearing behaviors in previous studies is that the range of fathers’ working hours was narrower in the countries where these studies were conducted and so did not clearly show differences between fathers with normal working hours and those with long working hours. The clear trend toward longer working hours in Japan offers a good opportunity to reexamine the impact of time constraints on fathers’ parenting behavior for a wider range of fathers’ working hours.

Against this backdrop, the purpose of this study was to address working hours as a major determinant of fathers’ parenting behavior and to examine its association with the frequency of parenting behaviors, using a dataset of about 43,000 father–mother pairs obtained from the Japan Environment and Children’s Study (JECS), a large cohort study in Japan.

## 2. Materials and methods

### 2.1. Study design

The JECS is a government-funded, nationwide birth cohort study that aims to evaluate the impact of environmental factors on children’s health and development. In total, 103,057 pregnancies were registered from 15 Regional Centres across Japan between January 2011 and March 2014, and detailed descriptions of the JECS can be found elsewhere (22). Mothers were recruited during pregnancy. The children’s fathers were recruited only after the mothers (or their children after birth) had started participation in the study. About half as many fathers as mothers were registered (23). Recruitment involved a face-to-face explanation of the survey, and written informed consent was obtained from all participants. The authors assert that all procedures contributing to the present work comply with the Helsinki Declaration of 1975, as revised in 2008. All procedures involving human subjects in the JECS protocol were reviewed and approved by the Ministry of the Environment’s Institutional Review Board on Epidemiological Studies (100910001) and the Ethics Committees of all participating institutions. The protocol of the present study was approved by the Institutional Review Board of the University of Toyama (R2020171).

### 2.2. Study data

**Figure 1:**
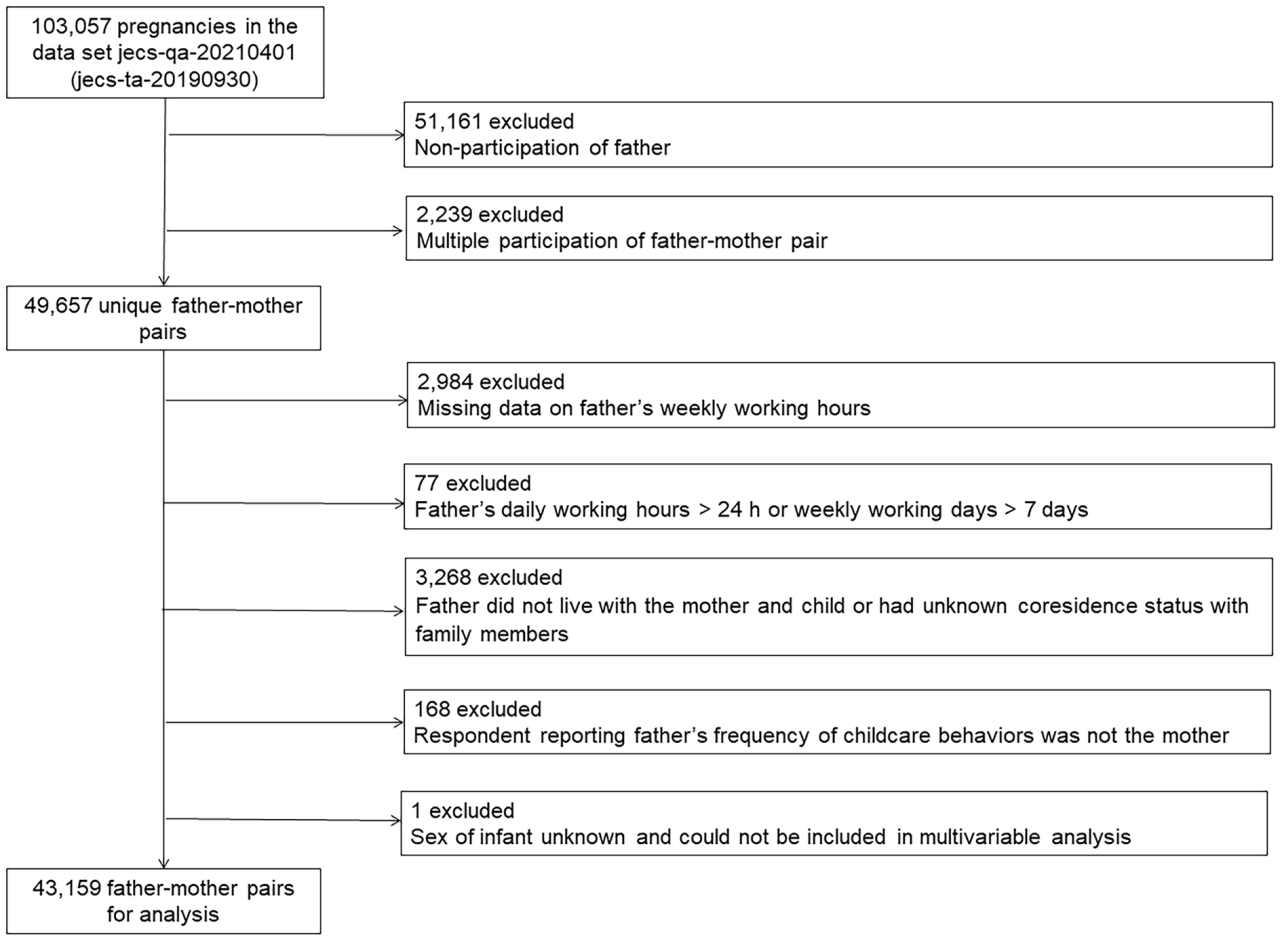
Participant flow diagram.

This study used the jecs-qa-20210401 (jecs-ta-20190930) dataset, which was first released in October 2019 and completed in February 2022. The dataset contained entries for 49,657 unique father–mother pairs. We excluded data without participation of the father (51,161) and with multiple participation of the father–mother pair (2,239). We also excluded pairs from the analysis for the following reasons: there was missing data on the father’s weekly working hours (2,984); the father’s daily working hours were >24 h or weekly working days were >7 days (77); the father did not live with the mother and child or the father’s coresidence status with family members was not known (3,268); the respondent reporting the father’s engagement in childcare behaviors was not the mother (168); and the infant’s sex was not known and thus could not be included in the multivariable analysis (1). Finally, data from 43,159 father–mother pairs were analyzed (Figure 1). In this study, we assumed that the father in each pair was the biological father of the child.

### 2.3. Measurements

A self-administered questionnaire was used to collect information on demographics, medical and obstetric history, physical and mental health status, lifestyle factors, occupation, and socioeconomic status. The questionnaire was distributed three times to mothers (during early and mid/late pregnancy and at 6 months after delivery) and once to fathers (between the mother’s pregnancy and when their infant reached 1 month of age). Data from the medical records were transcribed by physicians, midwives/nurses, and/or research co-ordinators.

### 2.4. Independent variables

The father’s weekly working hours was calculated from the “number of working days per week” and “number of working hours per day (including overtime)” obtained from the father’s survey responses. We then grouped working hours into six categories according to the classification of Tanaka et al. (24): ≥0 to ≤40 h; >40 to ≤45 h; >45 to ≤50 h; > 50 to ≤55 h; >55 to ≤65 h; and >65 h per week.

### 2.5. Dependent variables

Based on the mother’s survey responses at 6 months after delivery, the following seven parenting behaviors of the father were assessed: (a) playing at home, (b) playing outdoors, (c) helping with feeding, (d) changing diapers, (e) dressing, (f) bathing, and (g) putting the child to bed. Responses were provided on a 4-point scale (always, sometimes, rarely, and not at all). These seven items were selected for assessing the fathers’ parenting behaviors because similar items were assessed in previous studies in Japan, Western countries, and other Asian countries (25–28). Then, each of the fathers’ parenting behaviors was classified into a high-engagement group (always and sometimes) or a low-engagement group (rarely and never).

### 2.6. Covariates

The following covariates were considered: father’s age during the mother’s pregnancy (≤24 years; 25–29 years; 30–34 years; or ≥35 years); father’s highest educational level (junior high school or high school; technical junior college, technical/vocational college, or associate degree; or bachelor’s degree or graduate degree [master’s/doctorate]); father’s alcohol intake during the mother’s pregnancy (never-drinker; ex-drinker; current drinker); father’s smoking status during the mother’s pregnancy (never; previously did, but quit before learning of the mother’s pregnancy; previously did, but quit after learning of the mother’s pregnancy; currently smoking); annual household income (<4 million Japanese Yen [JPY]; 4 to <6 million JPY; or ≥6 million JPY); coresident family members of the father–mother pair during the pregnancy [twin or more of the surveyed infant(s); older sibling(s) of the surveyed infant(s); mother’s parent(s); or mother’s parent(s)-in-law]; the year the mother became pregnant (≤ 2011; 2012; ≥ 2013); and infant’s sex (male or female). In this study, the short form of the Autism-Spectrum Quotient Japanese version [AQ-J-10; (29)] was included to evaluate whether the father exhibited autistic traits during the mother’s pregnancy. The AQ-J-10 is a short form of the AQ (30) used to screen adolescents and adults with high-functioning pervasive developmental disorders and is a reliable and validated instrument. In this study, a score of ≥7 used as a cut-off (29) to categorize the results. Mother’s postpartum depression at 1 month after delivery was assessed using the Edinburgh Postnatal Depression Scale (EPDS), with a score of ≥9 used as the cut-off (31, 32) to categorize results. Cronbach’s alpha for the EPDS was 0.82, as shown in our previous study (33).

### 2.7. Statistical analysis

To estimate the risk of low engagement in fathers’ childcare behavior according to their weekly working hours category, we performed multivariable logistic regression analysis to calculate odds ratios (ORs) and 95% confidence intervals (CIs). Two-sided *p-*values less than 0.05 were considered to indicate statistical significance. Missing data of covariates were included as categorical variables in the adjusted model. In tests for trend, fathers’ weekly working hours was evaluated as a continuous variable. For the sensitivity analysis, we compared the adjusted ORs and 95% CIs obtained from categorizing fathers’ engagement in parenting behavior as “high” or “low” in the multivariable logistic regression analysis with those obtained from categorizing fathers’ engagement in parenting behavior according to four frequencies (always, sometimes, rarely, and never) in the multinomial logistic regression analysis. Data were analyzed using SAS version 9.4 software (SAS Institute Inc., Cary, NC).

## 3. Results

Table 1 shows the participants’ characteristics. In terms of hours worked, about 60% of fathers reported working ≤50 h per week, followed by 9.6% reporting working 50 h to ≤55 h, 16.2% reporting 55 h to ≤65 h, and 15.2% reporting >65 h. About 70% of the fathers were in their 30s or older. The most frequent highest educational level of the fathers was junior high school or high school (40.8%). Just under 70% of the respondents had household incomes of <6 million yen. In terms of family coresidence during the mother’s pregnancy, 51.7% of the father–mother pairs lived with the infant’s older sibling, 9.5% with the mother’s parent(s), and 12.4% with the mother’s parent(s)-in-law. Male sex of the infant was slightly more common (51.0%). The proportion of families raising multiple birth infants was 0.9%. During the mother’s pregnancy, 75.1% of fathers continued to drink and 41.0% continued to smoke. Autistic traits were reported in 6.6% of the fathers. The percentages of years in which mothers were pregnant was 35.0% before 2011, 33.3% in 2012, and 31.6% after 2013, almost the same percentage. Meanwhile, 14.3% of mothers showed symptoms of postpartum depression.

**Table 1:** Participant characteristics (*N* = 43,159).

Characteristics	*n* (%)
**Fathers’ weekly working hours category**	
≥0 to ≤40 h	9,328 (21.6)
>40 to ≤45 h	5,744 (13.3)
>45 to ≤50 h	10,414 (24.1)
>50 to ≤55 h	4,155 (9.6)
>55 to ≤65 h	6,979 (16.2)
>65 h	6,539 (15.2)
**Father’s age (years)**	
≤24	2,644 (6.1)
25–29	10,105 (23.4)
30–34	14,355 (33.3)
≥35	15,963(37.0)
Missing	92 (0.2)
**Father’s highest educational level**	
Junior high school or high school	17,599 (40.8)
Technical junior college, technical/vocational college, or associate degree	10,214 (23.7)
Bachelor’s degree, graduate degree (master’s/doctorate)	14,952 (34.6)
Missing	394 (0.9)
**Father’s alcohol intake during mother’s pregnancy**	
Never drank	9,095 (21.1)
Ex-drinker	1,511 (3.5)
Current drinker	32,425 (75.1)
Missing	128 (0.3)
**Father’s smoking status at mother’s pregnancy**	
Never	12,561 (29.1)
Previously did, but quit before learning of mother’s pregnancy	10,176 (23.6)
Previously did, but quit after learning of mother’s pregnancy	2,053 (4.8)
Currently smoking	17,696 (41.0)
Missing	673 (1.6)
**Father’s AQ-J-10 score during mother’s pregnancy**	
0–6	40,182 (93.1)
≥7	2,839 (6.6)
Missing	138 (0.3)
**Annual household income (JPY)**	
<4 million	15,375 (35.6)
4 to <6 million	13,856 (32.1)
≥6 million	11,270 (26.1)
Missing	2,658 (6.2)
**Coresident family members of the father–mother pair**	
Twin or more of the surveyed infant(s), yes	395 (0.9)
Older sibling(s) of the surveyed infant(s), yes	22,305 (51.7)
Mother’s parent(s), yes	4,096 (9.5)
Mother’s parent(s)-in-law, yes	5,358 (12.4)
**Year the mother became pregnant**	
≤2011	15,109 (35.0)
2012	14,351 (33.3)
≥2013	13,625 (31.6)
Missing	74 (0.2)
**Mother’s postpartum depression at 1 month after delivery** ^a^	
Yes	6,163 (14.3)
Missing	713 (1.7)
**Infant’s sex**	
Male	22,015 (51.0)
Female	21,144 (49.0)

AQ-J-10, Short form of the Autism-Spectrum Quotient Japanese version; JPY, Japanese Yen. ^a^Postpartum depression: total Edinburgh Postnatal Depression Scale score of ≥9.

Table 2 shows ORs and 95% CIs for the association between fathers’ weekly working hours and their engagement in parenting behavior when their child was 6 months old. After adjustment for covariates, fathers’ weekly working hours was still inversely associated with the frequency of all seven parenting behaviors examined in this study (*p* for trend <0.0001). Compared with fathers who worked ≥0 to ≤40 h per week, fathers with weekly working hours of >40 to ≤45 h had significantly higher adjusted ORs for low engagement in helping with feeding and bathing. Moreover, among fathers in the four remaining categories with weekly working hours of >45 h, the ORs for low engagement were significantly higher for all seven of the parenting behaviors. Among fathers who worked >45 to ≤50 h, adjusted ORs (95% CI) ranged from 1.10 (1.04–1.16; putting the child to bed) to 1.38 (1.21–1.57; playing at home). These ranged from 1.15 (1.06–1.26; dressing) to 1.38 (1.16–1.63; playing at home) among fathers who worked >50 to ≤55 h, and from 1.29 (1.21–1.37; putting the child to bed) to 1.65 (1.43–1.89, playing at home) among those who worked >55 to ≤65 h. Among fathers who worked >65 h per week, the ORs for low engagement in “playing at home” tended to be higher than for other parenting behaviors when compared with fathers who worked ≥0 to ≤40 h per week (adjusted OR [95%CI]: 2.38 [2.08–2.72]).

**Table 2:** Odds ratios (95% confidence intervals) for fathers’ low engagement in parenting behaviors when their infant(s) was 6 months old according to fathers’ weekly working hours category.

Engagement in paternal parenting behaviors	Fathers’ weekly working hours category						
	Group 0 ≥ 0 to ≤40 h	Group 1 > 40 to ≤45 h	Group 2 45 to ≤50 h	Group 3 > 50 to ≤55 h	Group 4 > 55 to ≤65 h	Group 5 >65 h	*p*-Value For trend
**Play**							
At home (*n* = 43,009)							
High	8,912 (95.8)	5,460 (95.4)	9,786 (94.4)	3,908 (94.4)	6,490 (93.3)	5,906 (90.7)	
Low	388 (4.2)	265 (4.6)	586 (5.7)	234 (5.7)	466 (6.7)	608 (9.3)	
Crude OR	1.00	1.12 (0.95–1.31)	**1.38 (1.21–1.57)**	**1.38 (1.16–1.63)**	**1.65 (1.44–1.89)**	**2.37 (2.07–2.70)**	**<0.0001**
Adjusted OR^a^	1.00	1.16 (0.99–1.37)	**1.38 (1.21–1.57)**	**1.38 (1.16–1.63)**	**1.65 (1.43–1.89)**	**2.38 (2.08–2.72)**	**<0.0001**
Outdoors (*n* = 42,995)							
High	7,107 (76.5)	4,340 (75.8)	7,658 (73.9)	3,036 (73.3)	4,990 (71.8)	4,457 (68.4)	
Low	2,183 (23.5)	1,386 (24.2)	2,711 (26.2)	1,106 (26.7)	1,962 (28.2)	2,059 (31.6)	
Crude OR	1.00	1.04 (0.96–1.12)	**1.15 (1.08–1.23)**	**1.19 (1.09–1.29)**	**1.28 (1.19–1.37)**	**1.50 (1.40–1.62)**	**<0.0001**
Adjusted OR^a^	1.00	1.05 (0.97–1.13)	**1.14 (1.07–1.22)**	**1.18 (1.08–1.28)**	**1.26 (1.17–1.35)**	**1.49 (1.39–1.60)**	**<0.0001**
**Caregiving**							
Helping with feeding (*n* = 42,838)							
High	6,624 (71.6)	3,918 (68.7)	6,915 (66.9)	2,710 (65.6)	4,336 (62.6)	3,810 (58.7)	
Low	2,630 (28.4)	1,785 (31.3)	3,417 (33.1)	1,420 (34.4)	2,595 (37.4)	2,678 (41.3)	
Crude OR	1.00	**1.15 (1.07–1.23)**	**1.25 (1.17–1.32)**	**1.32 (1.22–1.43)**	**1.51 (1.41–1.61)**	**1.77 (1.66–1.89)**	**<0.0001**
Adjusted OR^a^	1.00	**1.13 (1.05–1.21)**	**1.23 (1.15–1.30)**	**1.31 (1.21–1.42)**	**1.47 (1.37–1.57)**	**1.73 (1.62–1.86)**	**<0.0001**
Changing diapers (*n* = 43,021)							
High	7,658 (82.3)	4,722 (82.4)	8,171 (78.8)	3,221 (77.8)	5,254 (75.5)	4,531 (69.5)	
Low	1,643 (17.7)	1,006 (17.6)	2,202 (21.2)	920 (22.2)	1,702 (24.5)	1,991 (30.5)	
Crude OR	1.00	0.99 (0.91–1.08)	**1.26 (1.17–1.35)**	**1.33 (1.22–1.46)**	**1.51 (1.40–1.63)**	**2.05 (1.90–2.21)**	**<0.0001**
Adjusted OR^a^	1.00	1.04 (0.95–1.13)	**1.24 (1.16–1.34)**	**1.33 (1.21–1.45)**	**1.49 (1.38–1.61)**	**2.04 (1.89–2.20)**	**<0.0001**
Dressing (*n* = 43,027)							
High	7,147 (76.9)	4,392 (76.7)	7,660 (73.8)	3,071 (74.1)	4,839 (69.6)	4,237 (65.0)	
Low	2,153 (23.2)	1,336 (23.3)	2,718 (26.2)	1,074 (25.9)	2,116 (30.4)	2,284 (35.0)	
Crude OR	1.00	1.01 (0.93–1.09)	**1.18 (1.10–1.26)**	**1.16 (1.07–1.26)**	**1.45 (1.35–1.56)**	**1.79 (1.67–1.92)**	**<0.0001**
Adjusted OR^a^	1.00	1.03 (0.95–1.11)	**1.17 (1.09–1.25)**	**1.15 (1.06–1.26)**	**1.44 (1.34–1.54)**	**1.78 (1.66–1.91)**	**<0.0001**
Bathing (*n* = 43,015)							
High	8,109 (87.2)	4,912 (85.8)	8,734 (84.2)	3,436 (83.0)	5,693 (81.8)	5,023 (77.1)	
Low	1,194 (12.8)	812 (14.2)	1,636 (15.8)	706 (17.0)	1,265 (18.2)	1,495 (22.9)	
Crude OR	1.00	**1.12 (1.02–1.24)**	**1.27 (1.17–1.38)**	**1.40 (1.26–1.54)**	**1.51 (1.39–1.65)**	**2.02 (1.86–2.20)**	**<0.0001**
Adjusted OR^a^	1.00	**1.12 (1.02–1.23)**	**1.27 (1.17–1.37)**	**1.38 (1.25–1.53)**	**1.49 (1.36–1.62)**	**2.01 (1.84–2.18)**	**<0.0001**
Putting the child to bed (*n* = 43,002)							
High	4,900 (52.7)	2,942 (51.4)	5,174 (49.9)	2,000 (48.3)	3,176 (45.7)	2,780 (42.7)	
Low	4,400 (47.3)	2,784 (48.6)	5,191 (50.1)	2,143 (51.7)	3,780 (54.3)	3,732 (57.3)	
Crude OR	1.00	1.05 (0.99–1.13)	**1.12 (1.06–1.18)**	**1.19 (1.11–1.28)**	**1.33 (1.25–1.41)**	**1.50 (1.40–1.59)**	**<0.0001**
Adjusted OR^a^	1.00	1.04 (0.97–1.11)	**1.10 (1.04–1.16)**	**1.18 (1.10–1.27)**	**1.29 (1.21–1.37)**	**1.46 (1.37–1.56)**	**<0.0001**

^a^Covariates were adjusted for father’s age, father’s educational background, father’s alcohol intake at mother’s pregnancy, father’s smoking status at mother’s pregnancy, father’s autistic traits at mother’s pregnancy, annual household income, coresident family members of the mother–father pair [twin or more of the surveyed infant(s); older sibling(s) of the surveyed infant(s); mother’s parent(s); or mother’s parent(s)-in-law], year the mother became pregnant, mother’s postpartum depression at 1 month after delivery, infant’s sex. Bold indicates statistical significance (*p* < 0.05). OR, odds ratio.

Supplementary Table S1 shows adjusted ORs for each covariate in relation to the frequency of the fathers’ parenting behaviors. The OR for fathers with low engagement in all seven parenting behaviors was significantly higher when an older sibling(s) was present than when not present and when the mother showed symptoms of postpartum depression than when they did not. Furthermore, the ORs for fathers’ low engagement tended to be significantly higher in the following groups compared with the reference group: fathers aged ≥35 (reference group: ≤ 24), fathers who continued to smoke even after learning of the pregnancy (reference group: never), fathers with AQ-J-10 scores of ≥7 during the pregnancy (reference group: 0–6), fathers living with the mother’s parents (reference group: living elsewhere), and, interestingly, fathers with a female infant(s) (reference group: male).

Supplementary Table S2 shows the adjusted ORs and 95% confidence intervals obtained from categorizing fathers’ engagement in parenting behavior according to four frequencies (always, sometimes, rarely, and never) in the multinomial logistic regression analysis. The results were consistent with those obtained from categorizing fathers’ engagement in parenting behavior as “high” or “low” in the multivariable logistic regression analysis.

## 4. Discussion

This analysis of data from a large cohort study in Japan revealed a significant inverse association between fathers’ working hours and the frequency of their engagement in parenting behaviors. Fathers with the highest weekly working hours (> 65 h) showed a stronger tendency to have low engagement in the parenting behaviors of “playing at home,” “changing diapers,” and “bathing” compared with those who worked ≥0 to ≤40 h per week. In addition, this study classified fathers’ working hours per week into six groups to examine the association with the frequency of their parenting behaviors, thus allowing examination of the lower limit of weekly working hours associated with low-engagement parenting behaviors. Among the covariates considered, the following were associated with the fathers’ low engagement in parenting behavior: the infant(s) having an older sibling(s), the mother showing symptoms of postpartum depression, father’s age ≥ 35 years, father continuing to smoke even after learning of the pregnancy, father showing autistic traits, living with the mother’s parent(s), and female sex of the infant(s). These results suggest that the impact of fathers’ working hours on the frequency of their parenting behavior is large compared with the other variables included as covariates.

Many previous studies conducted outside Japan examining the association between fathers’ working hours and the frequency of parenting behaviors have found inverse associations similar to those found in this study, but findings on the strength of the association have been inconsistent. As indicated earlier in the introduction, it is possible that working hours considered long in previous studies outside of Japan were shorter than those in Japan; for example, Coles et al. (12) defined long working hours as ≥45 h per week and McGill (9) as ≥51 h per week. It is also possible that the distribution of working hours was not as wide and uniformly distributed as in Japan. Because of this, this study could examine the relationship between the frequency of fathers’ engagement in parenting behaviors and their working hours over a wider range of working hours compared with previous studies outside Japan. Previous studies conducted in Japan have generally reported inverse associations, but the number of subjects was several hundred to several thousand participants (17, 18). Therefore, to our knowledge, this is the first study to examine the relationship between these two factors among tens of thousands of participants in Japan. In addition, the study was able to consider a large number of covariates. With the strengths listed above, this study provides new findings supporting the inverse association found between parenting behaviors and fathers’ working hours.

The status of fathers’ parenting behaviors in each working hours category was determined in detail. First, fathers working >40 h to ≤45 h tended to be less engaged in helping with feeding and bathing compared with those working ≥0 to ≤40 h. The tendency to have low engagement in helping with feeding and bathing seems to be due to the fact that fathers with longer working hours come home late and so are not present when their children need to be fed or bathed. Fathers working >45 to ≤50 h were found to have lower engagement in all seven parenting behaviors evaluated in this study compared with those working ≥0 to ≤40 h. In Japan, a maximum amount of overtime work is set by law (which came into effect in April 2019 for large enterprises and April 2020 for small and medium-sized enterprises), which in principle may not exceed 45 h per month (34). A father working >45 to ≤50 h per week (i.e., > 5 h to ≤10 h of overtime per week) would fall below this monthly limit of 45 h per month. Among fathers in this study who worked longer (> 50 h per week), all the parenting behaviors examined showed low engagement, suggesting that weekly working hours of >45 to ≤50 h could be a guide for encouraging fathers’ participation in childcare.

Working >65 h per week corresponds to about 5 h of overtime per day. About one in six fathers in our data worked this much. Fathers in this group showed a stronger tendency to have low engagement in all parenting behaviors compared with fathers working ≥0 to ≤40 h per week. This suggests that time constraints due to long working hours are a major obstacle to fathers’ engagement in parenting behaviors. The data used in this study were obtained in the early 2010s. A survey conducted by the Ministry of Internal Affairs and Communications during the same period reported that 13.7% of men worked ≥60 h per week (35). Although the rate that we report here is slightly higher, it is presumably because the survey conducted by the Ministry included the entire working population aged ≥15, whereas the data in our study covered mainly people in their 20s–30s. Therefore, it is reasonable to assume that the data obtained in this study generally reflect the trend in working hours in Japan in the early 2010s.

It is worth noting that fathers’ long working hours is likely to have a strong negative effect on the time they engage in play, and the group that worked >65 h per week showed a trend toward higher ORs for low engagement, especially for “playing at home,” compared with other parenting behaviors. Fathers playing with their children has been reported to have a positive impact on children’s behavioral outcomes (36). The fact that working long hours was more strongly associated with low engagement in “playing at home” behavior compared with other parenting behaviors is presumably due to aspects of the business culture in Japan. From the time the data were collected to the present, most men in Japan have worked full-time with fixed starting hours (37, 38). Generally, an 8-h workday would consist of arriving at work at 9:00 a.m. and leaving at 5:00 p.m. On workdays, fathers are more likely to play with their children at home during the few leisure hours in the evening and night when the children are awake. Therefore, longer work hours would cause the father to return home later and likely have less leisure time. In this context, the more immediate aspects of childcare such as caregiving may take precedence over play at home. For example, “helping with feeding” and “dressing” might have been done before work hours, and thus might have been less affected by working hours compared with “play at home.”

Previous studies have indicated that long working hours have negative effects on both physical and mental health (39, 40). Takehara et al. surveyed 3,514 families with children under 1 year of age and reported that 11.0% of fathers had self-reported moderate or severe psychological distress (41). Previous studies outside Japan have also reported that “new fathers” attempt to balance their roles as breadwinners and childcare providers by reducing their leisure time (9). The long working hours seen in the present study could themselves have a negative influence on both physical and mental health, and the additional burden of childcare could further increase the risk of ill health. Currently, fathers are forced to balance childcare while maintaining long working hours, which is presumed to place a heavy burden on them individually.

Among the covariates considered, this study also found that fathers tended to be less engaged in childrearing when the children were girls than when they were boys. In early childhood, parents are considered to play an important role in socializing children into gender roles (42). It is theorized that fathers are more involved in the child-rearing of boys than girls because of shared interests, the desire to serve as a male role model, and the aspiration to invest in boys’ future educational and economic success, in line with traditional gender norms (43, 44). Therefore, even at 6 months of age, these fathers may be more involved with boys than girls because of these fathers’ mentalities.

As mentioned in the previous paragraph, according to a more recent survey conducted by the Ministry of Internal Affairs and Communications in 2021, the percentage of men working ≥60 h per week was 7.9% compared with 13.7% in 2011, indicating a downward trend (35, 45). This decline in long working hours reflects the effects of the “work-style reform” that the Japanese government has been promoting as policy. This suggests the effectiveness of efforts to reduce long working hours at the community and population level. The father’s employer and co-workers, who often form his closest social circle, may be able to play a role in generating time for him to engage in parenting behavior, as described below. To this end, it is necessary to change the environment that creates long working hours in the first place. Second, flexible working patterns need to be introduced. In 2021, only about 10% of men in Japan had flexible work arrangements with no fixed starting time (38), indicating that there is much room for improvement here. Third, it is important to establish a system that enables men with children to fully utilize the parental leave system. Revisions to labor laws in recent years have granted men more flexibility in terms of parental leave (46). However, there are still some barriers to realizing a work environment in which more men can take advantage of these systems. The actual effects of efforts to increase fathers’ engagement in parenting behaviors will need to be examined further in newer cohorts.

This study has three main strengths. First, reliable findings were obtained using data from a large cohort study in Japan to examine the effects of the fathers’ long working hours in relation to the frequency of their parenting behaviors, with adjustment for other factors. Second, we prospectively examined the relationship between their working hours and the frequency of their parenting behaviors and found a dose–response relationship. Third, the fathers’ parenting status was data obtained when their infant was 6 months old, so it was possible to examine data that was well controlled with respect to the infant’s age and the mother’s employment status (most mothers were presumed to be on childcare leave as provided in law).

This study also has some limitations. The first was the objectivity of the data on the frequency of the fathers’ parenting behaviors, which were assessed subjectively by the mothers. Previous studies have reported that wives tend to underestimate the frequency of their husbands’ parenting behavior (47). In addition, mother’s marital satisfaction may have influenced the subjective assessment of the father’s parenting behavior (48); however, this study did not obtain such data and so this potential factor could not be examined. Therefore, it is necessary to take into account the influence of bias due to the mothers’ subjective evaluation. The second limitation was the recruitment method. This study included only fathers whose partner had begun participation in the JECS and had consented to recruitment of the fathers. Fathers were recruited through a face-to-face method, but only about half as many fathers registered compared with mothers. This could have caused selection bias in which fathers who work in a way that prevented them from being present at the time of face-to-face recruitment were not included in the study. In addition, we cannot exclude the possibility that the father registered during the mother’s pregnancy was not the same father who was the subject of the evaluation of the frequency of parenting behaviors when the child was 6 months old, though such differences were considered to be very rare. The third limitation is the generalizability of the results obtained in this study. Fathers’ parenting behavior is also influenced by social frameworks such as traditional gender ideology that are deeply rooted in their society and by public childrearing support systems (15, 16). In the early 2010s when the data for this study was collected, parental leave systems for fathers were not as widespread outside of Europe and the United States (49). This could have been one reason why the relationship between fathers’ working hours and parenting behavior became clearer in this study. Further studies should be conducted in other countries and regions where men work longer hours, as in Japan, to see if a similar relationship is found between fathers’ longer working hours and their frequency of parenting behaviors. In addition, in recent years, government policies have expanded childcare support in Japan, for example, the law was revised to promote men taking parental leave (46). Therefore, it will be necessary to investigate over time the impact of these changes in public social systems on the relationship between fathers’ working hours and parenting behavior in Japan. The fourth limitation was that this observational study could not assess causal relationships. In Japan, as mentioned earlier, work-style reform is being promoted and long working hours are on the decline. In addition, the average time that men spent with a child under the age of 6 years on childcare increased in 2021 compared with 2011 (50, 51). This increase suggests that measures to decrease long working hours could be effective for increasing fathers’ parenting time, although a causal relationship cannot be determined.

## 5. Conclusion

This study found a significant inverse association between fathers’ working hours and the frequency of their parenting behaviors. Our results suggest that greater time constraints imposed by longer working hours are a major factor that discourages fathers from engaging in childrearing behavior. Intervention targeting long working hours could contribute to measures aimed at promoting high-engagement parenting behaviors among fathers. Since these data were obtained, measures aimed at reducing long working hours have been promoted as policy in Japan. However, the actual effects of these efforts to increase fathers’ engagement in parenting behaviors will need to be examined further in newer cohorts.

## Data availability statement

The data analyzed in this study is subject to the following licenses/restrictions: Data are unsuitable for public deposition due to ethical restrictions and the legal framework of Japan. Publicly depositing data containing personal information is prohibited by the Act on the Protection of Personal Information (Act No. 57 of 30 May 2003; amended 9 September 2015). The open sharing of epidemiologic data is also restricted by the Ethical Guidelines for Medical and Health Research Involving Human Subjects enforced by the Japan Ministry of Education, Culture, Sports, Science and Technology and the Ministry of Health, Labour and Welfare. All inquiries about access to data should be sent to: jecs-en@nies.go.jp. The person responsible for handling enquiries sent to this e-mail address is Dr. Shoji F. Nakayama, JECS Programme Office, National Institute for Environmental Studies. Requests to access these datasets should be directed to jecs-en@nies.go.jp.

## Ethics statement

The studies involving human participants were reviewed and approved by all procedures involving human subjects in the JECS protocol were reviewed and approved by the Ministry of the Environment’s Institutional Review Board on Epidemiological Studies (100910001) and the ethics committees of all participating institutions. This study protocol was approved by the Institutional Review Board of the University of Toyama. Written informed consent to participate in this study was provided by the participants’ legal guardian/next of kin.

## Author contributions

HK, AT, and HI conceived of and designed the study. HK and KM analyzed the data. HK drafted the manuscript. AT, KM, KH, MI, HI, and the JECS group critically reviewed the draft and checked the analysis. The JECS group collected the data and obtained the funding. AT, KM, and HI provided administrative, technical, and material support. All authors approved the submission of the manuscript in its current form.

## Funding

The JECS was funded by the Ministry of the Environment, Japan. The funding source played no role in the study’s design, collection, analysis, or interpretation of data; in the writing of the report; or in the decision to submit this paper for publication. The findings and conclusions of this article are solely the responsibility of the authors and do not represent the official views of the above government agency.

## Conflict of interest

The authors declare that the research was conducted in the absence of any commercial or financial relationships that could be construed as a potential conflict of interest.

## Publisher’s note

All claims expressed in this article are solely those of the authors and do not necessarily represent those of their affiliated organizations, or those of the publisher, the editors and the reviewers. Any product that may be evaluated in this article, or claim that may be made by its manufacturer, is not guaranteed or endorsed by the publisher.

## Appendix

Members of the JECS Group as of 2022: Michihiro Kamijima (Principal Investigator, Nagoya City University, Nagoya, Japan), Shin Yamazaki (National Institute for Environmental Studies, Tsukuba, Japan), Yukihiro Ohya (National Center for Child Health and Development, Tokyo, Japan), Reiko Kishi (Hokkaido University, Sapporo, Japan), Nobuo Yaegashi (Tohoku University, Sendai, Japan), Koichi Hashimoto (Fukushima Medical University, Fukushima, Japan), Chisato Mori (Chiba University, Chiba, Japan), Shuichi Ito (Yokohama City University, Yokohama, Japan), Zentaro Yamagata (University of Yamanashi, Chuo, Japan), Hidekuni Inadera (University of Toyama, Toyama, Japan), Takeo Nakayama (Kyoto University, Kyoto, Japan), Tomotaka Sobue (Osaka University, Suita, Japan), Masayuki Shima (Hyogo Medical University, Nishinomiya, Japan), Hiroshige Nakamura (Tottori University, Yonago, Japan), Narufumi Suganuma (Kochi University, Nankoku, Japan), Koichi Kusuhara (University of Occupational and Environmental Health, Kitakyushu, Japan), and Takahiko Katoh (Kumamoto University, Kumamoto, Japan).
