# Supplementary material for: Impact of longer working hours on fathers’ parenting behavior when their infants are 6 months old: The Japan Environment and Children’s Study
Source: Front Public Health. 2023 Jun 27;11:1100923. doi: 10.3389/fpubh.2023.1100923 (PMC10334997; doi:10.3389/fpubh.2023.1100923)
Supplement: Supplementary file 1 [file Table_1.docx]

Supplementary material

Impact of longer working hours on fathers’ parenting behavior when their infants are 6 months old: The Japan Environment and Children’s Study

Haruka Kasamatsu^1^, Akiko Tsuchida^1,2^, Kenta Matsumura^1,2^, Kei Hamazaki^1,2,3^, Mariko Inoue^2^, Hidekuni Inadera^1,2^*, and the Japan Environment and Children’s Study Group**

*** Correspondence**: Hidekuni Inadera: inadera@med.u-toyama.ac.jp

# Supplementary Tables

**Supplementary Table 1**. Adjusted odds ratios (95% confidence intervals) for low engagement in fathers’ parenting behaviors when their infant(s) was 6 months old according to covariates.

|  | Play | | Caregiving | | | | |
| --- | --- | --- | --- | --- | --- | --- | --- |
|  | At home | Outdoors | Helping with feeding | Changing diapers | Dressing | Bathing | Putting the child to bed |
|  | (n = 43,009) | (n = 42,995) | (n = 42,838) | (n = 43,021) | (n = 43,027) | (n = 43,015) | (n = 43,002) |
| Variable | Adjusted OR^a^ (95%CI) | Adjusted OR^a^ (95%CI) | Adjusted OR^a^ (95%CI) | Adjusted OR^a^ (95%CI) | Adjusted OR^a^ (95%CI) | Adjusted OR^a^ (95%CI) | Adjusted OR^a^  (95%CI) |
| Father’s age (years) |  |  |  |  |  |  |  |
| ≤ 24 | 1.00 | 1.00 | 1.00 | 1.00 | 1.00 | 1.00 | 1.00 |
| 25–29 | **0.72 (0.60–0.87)** | 1.00 (0.90–1.11) | 0.97 (0.88–1.07) | 0.94 (0.85–1.05) | 0.97 (0.87–1.07) | 0.96 (0.84–1.09) | 1.06 (0.97–1.16) |
| 30–34 | **0.77 (0.64–0.93)** | 1.11 (1.00–1.23) | **1.11 (1.01–1.22)** | 1.04 (0.94–1.16) | **1.19 (1.08–1.32)** | **1.15 (1.01–1.31)** | **1.09 (1.00–1.19)** |
| ≥ 35 | 0.90 (0.75–1.09) | **1.27 (1.15–1.41)** | **1.14 (1.03–1.25)** | **1.21 (1.09–1.35)** | **1.41 (1.27–1.56)** | **1.50 (1.32–1.70)** | 1.07 (0.98–1.17) |
| Missing | 0.98 (0.42–2.31) | 1.25 (0.78–1.99) | 1.26 (0.81–1.95) | 0.82 (0.47–1.43) | 0.83 (0.49–1.39) | 1.42 (0.82–2.44) | 1.02 (0.67–1.56) |
| Father’s highest educational level |  |  |  |  |  |  |  |
| Junior high school or  high school | 1.00 | 1.00 | 1.00 | 1.00 | 1.00 | 1.00 | 1.00 |
| Technical junior college,  technical/vocational  college, or associate  degree | 0.93 (0.83–1.04) | 0.95 (0.90–1.01) | 1.01 (0.96–1.06) | **0.85 (0.80–0.90)** | **0.90 (0.85–0.95)** | 1.04 (0.97–1.11) | 1.04 (0.99–1.09) |
| Bachelor’s degree,  graduate degree  (master’s/doctorate) | 1.09 (0.98–1.20) | 1.05 (1.00–1.11) | **1.20 (1.14–1.26)** | **0.94 (0.88–0.99)** | 1.00 (0.95–1.05) | **1.21 (1.14–1.29)** | **1.13 (1.08–1.19)** |
| Missing | **1.50 (1.03–2.19)** | 1.16 (0.92–1.47) | 1.01 (0.81–1.27) | 1.06 (0.83–1.36) | 1.03 (0.82–1.30) | 1.02 (0.77–1.35) | 1.09 (0.89–1.35) |
| Father’s alcohol intake at mother’s pregnancy |  |  |  |  |  |  |  |
| Never drank | 1.00 | 1.00 | 1.00 | 1.00 | 1.00 | 1.00 | 1.00 |
| Ex-drinker | 0.94 (0.75–1.19) | 0.98 (0.86–1.11) | 0.94 (0.84–1.06) | 1.04 (0.91–1.19) | 0.93 (0.82–1.06) | **1.16 (1.00–1.33)** | 0.93 (0.84–1.04) |
| Current drinker | 0.95 (0.86–1.05) | 1.02 (0.97–1.08) | 0.98 (0.93–1.03) | 0.97 (0.91–1.02) | 1.00 (0.95–1.06) | 0.95 (0.89–1.02) | 1.04 (0.99–1.09) |
| Missing | 1.46 (0.81–2.64) | 1.09 (0.74–1.61) | 1.31 (0.92–1.88) | 1.10 (0.74–1.66) | 1.07 (0.73–1.58) | 1.25 (0.81–1.94) | 1.40 (0.98–2.01) |
| Father’s smoking status at mother’s pregnancy |  |  |  |  |  |  |  |
| Never | 1.00 | 1.00 | 1.00 | 1.00 | 1.00 | 1.00 | 1.00 |
| Previously did, but quit  before realizing mother’s pregnancy | 1.01 (0.89–1.13) | 0.94 (0.89–1.00) | 0.95 (0.90–1.01) | 1.02 (0.95-1.09) | 0.96 (0.91-1.02) | **0.92 (0.86–0.99)** | **1.06 (1.01–1.12)** |
| Previously did, but quit  after realizing mother’s pregnancy | 1.01 (0.82–1.24) | 1.00 (0.89–1.11) | **0.88 (0.80–0.98)** | 0.91 (0.80–1.03) | **0.85 (0.76–0.95)** | 0.90 (0.79–1.03) | 1.00 (0.91–1.10) |
| Currently smoking | **1.19 (1.07–1.32)** | **1.12 (1.06–1.19)** | **1.17 (1.11–1.23)** | **1.45 (1.37–1.54)** | **1.19 (1.13–1.26)** | 1.04 (0.97–1.11) | **1.24 (1.18–1.30)** |
| Missing | 1.14 (0.83–1.56) | 1.18 (0.99–1.40) | 1.11 (0.94–1.31) | 1.11 (0.92–1.34) | 1.00 (0.84–1.20) | 1.00 (0.81–1.24) | 1.09 (0.93–1.27) |
| Father's AQ-J-10 score during mother’s pregnancy |  |  |  |  |  |  |  |
| 0–6 | 1.00 | 1.00 | 1.00 | 1.00 | 1.00 | 1.00 | 1.00 |
| ≥7 | **1.37 (1.18–1.59)** | 1.06 (0.98–1.16) | 1.05 (0.97–1.14) | **1.10 (1.00–1.20)** | 1.07 (0.98-1.17) | **1.19 (1.08–1.32)** | 1.08 (1.00–1.17) |
| Missing | **1.96 (1.16–3.31)** | 1.23 (0.85–1.78) | 1.27 (0.89–1.80) | **1.81 (1.26–2.61)** | **1.66 (1.17–2.36)** | **1.53 (1.02–2.29)** | 0.98 (0.70–1.38) |
| Annual household income (JPY) |  |  |  |  |  |  |  |
| < 4 million | 1.00 | 1.00 | 1.00 | 1.00 | 1.00 | 1.00 | 1.00 |
| 4 to < 6 million | 0.90 (0.82-1.00) | **0.93 (0.88–0.99)** | **1.11 (1.05–1.16)** | **0.92 (0.87–0.98)** | 1.00 (0.95–1.06) | **0.89 (0.83–0.94)** | 1.04 (0.99–1.09) |
| ≥ 6 million | 0.97 (0.87-1.08) | 0.97 (0.92-1.03) | **1.19 (1.13–1.26)** | **0.91 (0.86–0.97)** | 1.00 (0.94–1.06) | **0.84 (0.78–0.90)** | **1.12 (1.06–1.18)** |
| Missing | 0.96 (0.80-1.15) | 0.95 (0.86-1.04) | 0.99 (0.90–1.09) | 0.95 (0.86–1.06) | 1.01 (0.91–1.11) | 0.91 (0.80–1.02) | 0.95 (0.87–1.03) |
| Co-resident family members |  |  |  |  |  |  |  |
| Surveyed infant(s) |  |  |  |  |  |  |  |
| Single | 1.00 | 1.00 | 1.00 | 1.00 | 1.00 | 1.00 | 1.00 |
| Twin or more | 1.21 (0.81–1.82) | 1.02 (0.82–1.28) | **0.51 (0.40–0.66)** | **0.61 (0.46–0.82)** | 1.04 (0.83–1.30) | 0.89 (0.67–1.18) | **0.35 (0.28–0.44)** |
| Living with older  sibling(s) of the surveyed infant(s) |  |  |  |  |  |  |  |
| No | 1.00 | 1.00 | 1.00 | 1.00 | 1.00 | 1.00 | 1.00 |
| Yes | **2.71 (2.47–2.97)** | **1.31 (1.25–1.37)** | **1.12 (1.07–1.17)** | **1.58 (1.50–1.66)** | **1.14 (1.09–1.19)** | **1.39 (1.32–1.47)** | **1.10 (1.05–1.14)** |
| Living with mother’s parent(s) |  |  |  |  |  |  |  |
| No | 1.00 | 1.00 | 1.00 | 1.00 | 1.00 | 1.00 | 1.00 |
| Yes | **1.24 (1.08–1.42)** | 1.00 (0.93–1.08) | 1.01 (0.94–1.09) | **1.12 (1.04–1.22)** | **1.18 (1.10–1.27)** | **1.13 (1.04–1.24)** | **0.92 (0.86–0.98)** |
| Living with mother’s parent(s)-in-law |  |  |  |  |  |  |  |
| No | 1.00 | 1.00 | 1.00 | 1.00 | 1.00 | 1.00 | 1.00 |
| Yes | 0.98 (0.86–1.11) | 0.95 (0.89–1.01) | 0.96 (0.90–1.02) | **1.20 (1.12–1.28)** | **1.21 (1.13–1.29)** | **0.81 (0.75–0.88)** | 0.95 (0.89–1.01) |
| Year the mother became pregnant |  |  |  |  |  |  |  |
| ≤ 2011 | 1.00 | 1.00 | 1.00 | 1.00 | 1.00 | 1.00 | 1.00 |
| 2012 | 0.97 (0.88–1.07) | 0.97 (0.92–1.02) | **0.93 (0.89–0.98)** | 1.00 (0.94–1.05) | 1.01 (0.96–1.06) | **1.07 (1.01–1.14)** | 1.02 (0.97–1.06) |
| ≥ 2013 | 0.98 (0.89–1.08) | 0.98 (0.93–1.03) | 0.96 (0.91–1.01) | 0.99 (0.94–1.05) | 1.01 (0.96–1.07) | 1.06 (0.99-1.13) | 1.00 (0.95–1.05) |
| Missing | 1.49 (0.64–3.51) | 0.86 (0.50–1.48) | 0.85 (0.52–1.40) | 0.76 (0.41–1.44) | 1.10 (0.65–1.86) | 1.48 (0.83-2.63) | 1.15 (0.72–1.83) |
| Mother’s postpartum depression at 1 month after delivery^b^ |  |  |  |  |  |  |  |
| No | 1.00 | 1.00 | 1.00 | 1.00 | 1.00 | 1.00 | 1.00 |
| Yes | **1.88 (1.70–2.08)** | **1.32 (1.24–1.40)** | **1.15 (1.09–1.22)** | **1.22 (1.15–1.31)** | **1.37 (1.29–1.46)** | **1.17 (1.09–1.26)** | **1.07 (1.02–1.14)** |
| Missing | **1.36 (1.02–1.82)** | 0.98 (0.83–1.17) | 0.92 (0.78–1.09) | 0.91 (0.75–1.09) | 0.97 (0.82–1.15) | 0.93 (0.75–1.14) | 0.91 (0.78–1.06) |
| Infant’s sex |  |  |  |  |  |  |  |
| Male | 1.00 | 1.00 | 1.00 | 1.00 | 1.00 | 1.00 | 1.00 |
| Female | **1.09 (1.00–1.18)** | **1.05 (1.01–1.10)** | 1.04 (0.99–1.08) | 1.01 (0.96–1.06) | **1.06 (1.02–1.11)** | **1.13 (1.07–1.19)** | **1.15 (1.11–1.20)** |
|  |  |  |  |  |  |  |  |

*^a^Adjusted model included all of the above variables and fathers’ weekly working hours category.*

*^b^ Postpartum depression: total Edinburgh Postnatal Depression Scale score of ≥ 9.*

*Bold indicates statistical significance (p < .05).*

*Abbreviations: CI, confidence interval; OR, odds ratio; JPY, Japanese Yen.*

**Supplementary Table 2**. Odds ratios (95% confidence intervals) for each frequency (always, sometimes, rarely, not at all) of fathers’ engagement in parenting behaviors when their infant(s) was 6 months old according to fathers’ weekly working hours category.

|  |  | Fathers’ weekly working hours category | | | | | |  |
| --- | --- | --- | --- | --- | --- | --- | --- | --- |
| Engagement in paternal parenting behaviors | | Group 0 | Group 1 | Group 2 | Group 3 | Group 4 | Group 5 | p-value for trend |
|  |  | ≥ 0 to ≤ 40 h | > 40 to ≤ 45 h | > 45 to ≤ 50 h | > 50 to ≤ 55 h | > 55 to ≤ 65 h | > 65 h |  |
| **Play** | |  |  |  |  |  |  |  |
| At home | |  |  |  |  |  |  |  |
|  | Always | 5,326 (57.3) | 3,109 (54.3) | 5,379 (51.9) | 2,092 (50.5) | 3,195 (45.9) | 2,714 (41.7) |  |
|  | Sometimes | 3,586 (38.6) | 2,351 (41.1) | 4,407 (42.5) | 1,816 (43.8) | 3,295 (47.4) | 3,192 (49.0) |  |
|  | Crude OR | 1.00 | **1.12 (1.05–1.20)** | **1.22 (1.15–1.29)** | **1.29 (1.20–1.39)** | **1.53 (1.44–1.63)** | **1.75 (1.64–1.87)** | < .0001 |
|  | Adjusted OR^a^ | 1.00 | **1.12 (1.04–1.20)** | **1.21 (1.14–1.28)** | **1.29 (1.20–1.40)** | **1.53 (1.43–1.63)** | **1.77 (1.65–1.89)** | < .0001 |
|  | Rarely | 330 (3.6) | 225 (3.9) | 492 (4.7) | 200 (4.8) | 381 (5.5) | 510 (7.8) |  |
|  | Crude OR | 1.00 | 1.17 (0.98–1.39) | **1.48 (1.28–1.71)** | **1.54 (1.29–1.85)** | **1.93 (1.65–2.25)** | **3.03 (2.62–3.51)** | < .0001 |
|  | Adjusted OR^a^ | 1.00 | **1.21 (1.01–1.45)** | **1.48 (1.28–1.72)** | **1.56 (1.30–1.88)** | **1.95 (1.67–2.28)** | **3.14 (2.71–3.64)** | < .0001 |
|  | Not at all | 58 (0.6) | 40 (0.7) | 94 (0.9) | 34 (0.8) | 85 (1.2) | 98 (1.5) |  |
|  | Crude OR | 1.00 | 1.18 (0.79–1.77) | **1.61 (1.15–2.23)** | 1.49 (0.97–2.29) | **2.44 (1.75–3.42)** | **3.32 (2.39–4.60)** | < .0001 |
|  | Adjusted OR^a^ | 1.00 | 1.28 (0.85–1.92) | **1.61 (1.16–2.24)** | 1.51 (0.98–2.32) | **2.48 (1.76–3.47)** | **3.38 (2.43–4.71)** | < .0001 |
|  |  |  |  |  |  |  |  |  |
| Outdoors | |  |  |  |  |  |  |  |
|  | Always | 2,361 (25.4) | 1,360 (23.8) | 2,313 (22.3) | 830 (20.0) | 1,320 (19.0) | 1,111 (17.1) |  |
|  | Sometimes | 4,746 (51.1) | 2,980 (52.0) | 5,345 (51.6) | 2,206 (53.3) | 3,670 (52.8) | 3,346 (51.4) |  |
|  | Crude OR | 1.00 | **1.09 (1.01–1.18)** | **1.15 (1.07–1.23)** | **1.32 (1.20–1.45)** | **1.38 (1.28–1.50)** | **1.50 (1.38–1.63)** | < .0001 |
|  | Adjusted OR^a^ | 1.00 | 1.06 (0.98–1.15) | **1.13 (1.05–1.21)** | **1.31 (1.19–1.44)** | **1.35 (1.25–1.46)** | **1.47 (1.35–1.60)** | < .0001 |
|  | Rarely | 1,465 (15.8) | 926 (16.2) | 1,765 (17.0) | 748 (18.1) | 1,282 (18.4) | 1,284 (19.7) |  |
|  | Crude OR | 1.00 | 1.10 (0.99–1.22) | **1.23 (1.12–1.35)** | **1.45 (1.29–1.64)** | **1.57 (1.42–1.73)** | **1.86 (1.68–2.07)** | < .0001 |
|  | Adjusted OR^a^ | 1.00 | 1.08 (0.97–1.20) | **1.20 (1.10–1.32)** | **1.43 (1.27–1.61)** | **1.52 (1.37–1.68)** | **1.82 (1.64–2.02)** | < .0001 |
|  | Not at all | 718 (7.7) | 460 (8.0) | 946 (9.1) | 358 (8.6) | 680 (9.8) | 775 (11.9) |  |
|  | Crude OR | 1.00 | 1.11 (0.97–1.27) | **1.35 (1.20–1.51)** | **1.42 (1.22–1.65)** | **1.69 (1.50–1.92)** | **2.29 (2.03–2.60)** | < .0001 |
|  | Adjusted OR^a^ | 1.00 | 1.11 (0.97–1.27) | **1.32 (1.18–1.47)** | **1.39 (1.20–1.62)** | **1.64 (1.44–1.86)** | **2.24 (1.97–2.53)** | < .0001 |
|  |  |  |  |  |  |  |  |  |
| **Caregiving** | |  |  |  |  |  |  |  |
| Helping with feeding | |  |  |  |  |  |  |  |
|  | Always | 2,752 (29.7) | 1,463 (25.7) | 2,484 (24.0) | 947 (22.9) | 1,364 (19.7) | 1,117 (17.2) |  |
|  | Sometimes | 3,872 (41.8) | 2,455 (43.1) | 4,431 (42.9) | 1,763 (42.7) | 2,972 (42.9) | 2,693 (41.5) |  |
|  | Crude OR | 1.00 | **1.19 (1.10–1.29)** | **1.27 (1.18–1.36)** | **1.32 (1.21–1.45)** | **1.55 (1.43–1.68)** | **1.71 (1.57–1.87)** | < .0001 |
|  | Adjusted OR^a^ | 1.00 | **1.17 (1.08–1.27)** | **1.25 (1.16–1.34)** | **1.31 (1.20–1.44)** | **1.52 (1.40–1.65)** | **1.68 (1.55–1.83)** | < .0001 |
|  | Rarely | 1,514 (16.4) | 984 (17.3) | 1,852 (17.9) | 788 (19.1) | 1,392 (20.1) | 1,405 (21.7) |  |
|  | Crude OR | 1.00 | **1.22 (1.10–1.35)** | **1.36 (1.24–1.48)** | **1.51 (1.35–1.69)** | **1.86 (1.68–2.05)** | **2.29 (2.07–2.53)** | < .0001 |
|  | Adjusted OR^a^ | 1.00 | **1.19 (1.08–1.32)** | **1.32 (1.21–1.44)** | **1.50 (1.34–1.68)** | **1.80 (1.63–1.98)** | **2.23 (2.01–2.47)** | < .0001 |
|  | Not at all | 1,116 (12.1) | 801 (14.1) | 1,565 (15.2) | 632 (15.3) | 1,203 (17.4) | 1,273 (19.6) |  |
|  | Crude OR | 1.00 | **1.35 (1.21–1.51)** | **1.55 (1.41–1.71)** | **1.65 (1.46–1.86)** | **2.18 (1.96–2.41)** | **2.81 (2.53–3.13)** | < .0001 |
|  | Adjusted OR^a^ | 1.00 | **1.30 (1.17–1.46)** | **1.51 (1.38–1.66)** | **1.62 (1.43–1.83)** | **2.08 (1.88–2.31)** | **2.71 (2.44–3.02)** | < .0001 |
|  |  |  |  |  |  |  |  |  |
| Changing diapers | |  |  |  |  |  |  |  |
|  | Always | 3,005 (32.3) | 1,665 (29.1) | 2,820 (27.2) | 1,025 (24.8) | 1,493 (21.5) | 1,264 (19.4) |  |
|  | Sometimes | 4,653 (50.0) | 3,057 (53.4) | 5,351 (51.6) | 2,196 (53.0) | 3,761 (54.1) | 3,267 (50.1) |  |
|  | Crude OR | 1.00 | **1.19 (1.10–1.28)** | **1.23 (1.15–1.31)** | **1.38 (1.27–1.51)** | **1.63 (1.51–1.75)** | **1.67 (1.54–1.81)** | < .0001 |
|  | Adjusted OR^a^ | 1.00 | **1.17 (1.09–1.27)** | **1.21 (1.13–1.29)** | **1.37 (1.26–1.50)** | **1.59 (1.48–1.72)** | **1.65 (1.52–1.78)** | < .0001 |
|  | Rarely | 1,117 (12.0) | 710 (12.4) | 1,471 (14.2) | 632 (15.3) | 1,082 (15.6) | 1,261 (19.3) |  |
|  | Crude OR | 1.00 | **1.15 (1.03–1.28)** | **1.40 (1.28–1.54)** | **1.66 (1.47–1.87)** | **1.95 (1.76–2.16)** | **2.68 (2.42–2.98)** | < .0001 |
|  | Adjusted OR^a^ | 1.00 | **1.18 (1.05–1.32)** | **1.38 (1.25–1.51)** | **1.65 (1.46–1.86)** | **1.90 (1.71–2.11)** | **2.65 (2.38–2.94)** | < .0001 |
|  | Not at all | 526 (5.7) | 296 (5.2) | 731 (7.1) | 288 (7.0) | 620 (8.9) | 730 (11.2) |  |
|  | Crude OR | 1.00 | 1.02 (0.87–1.19) | **1.48 (1.31–1.68)** | **1.61 (1.37–1.88)** | **2.37 (2.08–2.71)** | **3.30 (2.90–3.76)** | < .0001 |
|  | Adjusted OR^a^ | 1.00 | 1.08 (0.93–1.26) | **1.45 (1.28–1.64)** | **1.60 (1.36–1.88)** | **2.32 (2.03–2.65)** | **3.28 (2.87–3.74)** | < .0001 |
|  |  |  |  |  |  |  |  |  |
| Dressing | |  |  |  |  |  |  |  |
|  | Always | 2,657 (28.6) | 1,465 (25.6) | 2,454 (23.7) | 925 (22.3) | 1,366 (19.6) | 1,132 (17.4) |  |
|  | Sometimes | 4,490 (48.3) | 2,927 (51.1) | 5,206 (50.2) | 2,146 (51.8) | 3,473 (49.9) | 3,105 (47.6) |  |
|  | Crude OR | 1.00 | **1.18 (1.09–1.28)** | **1.26 (1.17–1.34)** | **1.37 (1.25–1.50)** | **1.51 (1.39–1.63)** | **1.62 (1.49–1.76)** | < .0001 |
|  | Adjusted OR^a^ | 1.00 | **1.16 (1.07–1.26)** | **1.23 (1.15–1.32)** | **1.36 (1.24–1.49)** | **1.47 (1.36–1.59)** | **1.60 (1.47–1.74)** | < .0001 |
|  | Rarely | 1,485 (16.0) | 932 (16.3) | 1,810 (17.4) | 725 (17.5) | 1,357 (19.5) | 1,388 (21.3) |  |
|  | Crude OR | 1.00 | **1.14 (1.03–1.26)** | **1.32 (1.21–1.44)** | **1.40 (1.25–1.58)** | **1.78 (1.61–1.96)** | **2.19 (1.98–2.43)** | < .0001 |
|  | Adjusted OR^a^ | 1.00 | **1.13 (1.02–1.26)** | **1.29 (1.18–1.41)** | **1.39 (1.24–1.56)** | **1.74 (1.57–1.92)** | **2.17 (1.96–2.40)** | < .0001 |
|  | Not at all | 668 (7.2) | 404 (7.1) | 908 (8.8) | 349 (8.4) | 759 (10.9) | 896 (13.7) |  |
|  | Crude OR | 1.00 | 1.10 (0.96–1.26) | **1.47 (1.31–1.65)** | **1.50 (1.29–1.74)** | **2.21 (1.96–2.50)** | **3.15 (2.79–3.56)** | < .0001 |
|  | Adjusted OR^a^ | 1.00 | 1.12 (0.97–1.29) | **1.44 (1.28–1.61)** | **1.48 (1.27–1.72)** | **2.15 (1.90–2.43)** | **3.10 (2.75–3.51)** | < .0001 |
|  |  |  |  |  |  |  |  |  |
| Bathing | |  |  |  |  |  |  |  |
|  | Always | 5,130 (55.1) | 2,785 (48.7) | 4,685 (45.2) | 1,817 (43.9) | 2,595 (37.3) | 2,104 (32.3) |  |
|  | Sometimes | 2,979 (32.0) | 2,127 (37.2) | 4,049 (39.1) | 1,619 (39.1) | 3,098 (44.5) | 2,919 (44.8) |  |
|  | Crude OR | 1.00 | **1.32 (1.22–1.41)** | **1.49 (1.40–1.58)** | **1.53 (1.42–1.66)** | **2.06 (1.92–2.20)** | **2.39 (2.22–2.57)** | < .0001 |
|  | Adjusted OR^a^ | 1.00 | **1.28 (1.19–1.37)** | **1.48 (1.39–1.57)** | **1.54 (1.42–1.67)** | **2.04 (1.90–2.19)** | **2.40 (2.24–2.59)** | < .0001 |
|  | Rarely | 633 (6.8) | 423 (7.4) | 864 (8.3) | 392 (9.5) | 700 (10.1) | 816 (12.5) |  |
|  | Crude OR | 1.00 | **1.23 (1.08–1.40)** | **1.50 (1.34–1.67)** | **1.75 (1.53–2.01)** | **2.19 (1.94–2.46)** | **3.14 (2.80–3.53)** | < .0001 |
|  | Adjusted OR^a^ | 1.00 | **1.21 (1.06–1.38)** | **1.49 (1.33–1.66)** | **1.75 (1.53–2.01)** | **2.17 (1.92–2.44)** | **3.16 (2.82–3.56)** | < .0001 |
|  | Not at all | 561 (6.0) | 389 (6.8) | 772 (7.4) | 314 (7.6) | 565 (8.1) | 679 (10.4) |  |
|  | Crude OR | 1.00 | **1.28 (1.11–1.47)** | **1.51 (1.34–1.69)** | **1.58 (1.36–1.83)** | **1.99 (1.76–2.26)** | **2.95 (2.61–3.34)** | < .0001 |
|  | Adjusted OR^a^ | 1.00 | **1.27 (1.10–1.45)** | **1.50 (1.33–1.68)** | **1.57 (1.35–1.82)** | **1.95 (1.72–2.22)** | **2.95 (2.60–3.33)** | < .0001 |
|  |  |  |  |  |  |  |  |  |
| Putting the child to bed | |  |  |  |  |  |  |  |
|  | Always | 1,415 (15.2) | 812 (14.2) | 1,298 (12.5) | 529 (12.8) | 727 (10.5) | 606 (9.3) |  |
|  | Sometimes | 3,485 (37.5) | 2,130 (37.2) | 3,876 (37.4) | 1,471 (35.5) | 2,449 (35.2) | 2,174 (33.4) |  |
|  | Crude OR | 1.00 | 1.07 (0.96–1.18) | **1.21 (1.11–1.32)** | **1.13 (1.00–1.27)** | **1.37 (1.23–1.52)** | **1.46 (1.31–1.62)** | < .0001 |
|  | Adjusted OR^a^ | 1.00 | 1.06 (0.95–1.17) | **1.20 (1.10–1.31)** | 1.12 (1.00–1.26) | **1.34 (1.21–1.49)** | **1.43 (1.28–1.59)** | < .0001 |
|  | Rarely | 2,443 (26.3) | 1,591 (27.8) | 2,868 (27.7) | 1,163 (28.1) | 2,019 (29.0) | 1,883 (28.9) |  |
|  | Crude OR | 1.00 | **1.14 (1.02–1.26)** | **1.28 (1.17–1.40)** | **1.27 (1.13–1.44)** | **1.61 (1.45–1.79)** | **1.80 (1.61–2.01)** | < .0001 |
|  | Adjusted OR^a^ | 1.00 | 1.11 (1.00–1.24) | **1.25 (1.14–1.37)** | **1.26 (1.11–1.42)** | **1.55 (1.39–1.73)** | **1.74 (1.55–1.95)** | < .0001 |
|  | Not at all | 1,957 (21.0) | 1,193 (20.8) | 2,323 (22.4) | 980 (23.7) | 1,761 (25.3) | 1,849 (28.4) |  |
|  | Crude OR | 1.00 | 1.06 (0.95–1.19) | **1.29 (1.18–1.43)** | **1.34 (1.18–1.52)** | **1.75 (1.57–1.96)** | **2.21 (1.97–2.47)** | < .0001 |
|  | Adjusted OR^a^ | 1.00 | 1.04 (0.93–1.17) | **1.26 (1.14–1.39)** | **1.31 (1.16–1.49)** | **1.67 (1.50–1.87)** | **2.12 (1.89–2.37)** | < .0001 |
|  |  |  |  |  |  |  |  |  |

^a^ *Covariates were adjusted for father’s age, father’s educational background, father’s alcohol intake at mother’s pregnancy, father’s smoking status at mother’s pregnancy, father’s autistic traits at mother’s pregnancy, annual household income, coresident family members of the mother–father pair (twin or more of the surveyed infant(s); older sibling(s) of the surveyed infant(s); mother’s parent(s); or mother’s parent(s)-in-law), year the mother became pregnant, mother’s postpartum depression at 1 month after delivery, infant’s sex.*

*Bold indicates statistical significance (p < .05).*
